# Psychosocial Implications of COVID-19 on Head and Neck Cancer

**DOI:** 10.3390/curroncol29020090

**Published:** 2022-02-13

**Authors:** Sarah M. Dermody, Andrew G. Shuman

**Affiliations:** 1 Department of Otolaryngology–Head and Neck Surgery, University of Michigan Medical School, Ann Arbor, MI 48109, USA; sadermod@med.umich.edu; 2 Center for Bioethics and Social Sciences in Medicine, University of Michigan Medical School, Ann Arbor, MI 48109, USA; 3 Rogel Cancer Center, University of Michigan Medical School, Ann Arbor, MI 48109, USA

**Keywords:** COVID-19, ethics, medical, physician-patient relations, psycho-oncology, psychosocial intervention, head and neck neoplasms

## Abstract

The COVID-19 pandemic has fundamentally changed healthcare access, delivery, and treatment paradigms throughout oncology. Patients with head and neck cancer comprise an especially vulnerable population due to the nature of their disease and the transmission mechanism of the SARS-CoV-2 virus. The consequences of triage decisions and delays in care have serious psychosocial implications for patients. The development of structured psychosocial support programs, coupled with clear and consistent communication from treating physicians, can help mitigate perceptions of abandonment and distress that may accompany delays in care. As the unpredictability of the pandemic’s course continues to burden both providers and patients, we must be proactive in addressing the psychosocial implications of these delays in care.

## 1. Introduction

In March of 2020, the World Health Organization (WHO) declared the novel coronavirus disease 2019 (COVID-19), caused by the SARS-CoV-2 virus, a global pandemic [1]. Through this unprecedented time of rising death tolls and mutating variants, patients and providers have been impacted physically and psychosocially. With the introduction of effective COVID-19 vaccines and treatments ushering a new era of cautious optimism, we can garner important lessons that will outlast the current pandemic. Through the process of establishing systems to prioritize surgical care, the need to effectively communicate triage-level decisions to patients with respect for and acknowledgment of the psychosocial implications of delays in care has become clear.

Oncology patients are among those considered a highly vulnerable population due to their immunocompromised status [2]. The risk of death and severe complications from SARS-CoV-2 infection is increased in those with an underlying cancer diagnosis [3,4]. The heightened vulnerability of this patient population extends both to their physical and mental health, both critical components in comprehensive oncologic care. 

Head and neck cancer is particularly impacted by the pandemic in myriad ways. The centrality of the aerodigestive tract to its assessment and management increases the risk to both providers and patients. In addition, patients with altered anatomy and surgical airways may face challenges with masks and other barrier protections. Psychosocial consequences of the pandemic are also magnified in the population relating to higher rates of social isolation, communication challenges, and increased incidence of mental health disorders and substance dependence.

Equipment shortages, hospital capacity restrictions, and staffing limitations have forced health systems to continuously adapt their practices to suit the epidemiologic evolution of COVID-19 and its downstream impact. The routine management of oncology patients has evolved from an individualized approach to necessitating a public health ethics perspective, causing providers to face moral dilemmas regarding patient prioritization and triage. The distinction between clinical ethics, which involves the physician–patient relationship in developing individualized treatment plans to reach the best patient outcome, and public health ethics, which focuses on the needs of the population, is one that may be difficult for clinicians to accept [5,6]. Patients’ understanding of these concepts and their perceptions of their own care delivery must be recognized and addressed. 

## 2. Surgical Triage

A plethora of triage schemata have been developed throughout the pandemic, with some focusing specifically on surgical oncology patients [7,8,9,10]. For head and neck oncology, the American Academy of Otolaryngology–Head and Neck Surgery issued a position statement during the first wave of the pandemic that urged clinicians to defer all clinical interactions not deemed urgent or emergent [11]. Their position recognized the role of the individual physician in determining the urgency of each patient’s case—something that may be inherently subjective. The relative urgency of various head and neck cancers adds a layer of complexity to triage in this patient population and requires a nuanced understanding of the specialty. For example, an individual with a rapidly progressive laryngeal carcinoma demands a different level of urgency than a patient with a low-risk well-differentiated thyroid cancer. To address the complexities in surgical prioritization, an expert panel of international head and neck cancer surgeons developed the Surgical Prioritization and Rank Tool and Navigation Aid for Head and Neck Cancer (SPARTAN-HN) [8]. This surgical prioritization algorithm provides a framework for use in the COVID-19 era to stratify head and neck cancer patients requiring surgical care. 

As each patient’s clinical scenario is unique, their responses to delays in care or alterations of the planned treatment course will vary and may not always correlate with severity of disease. Patient reactions to these triage decisions may include anger, fear, apprehension, and abandonment. We must address the psychological consequences that surgical prioritization may have on patients who experience a waiting period due to triage decisions. The following recommendations provide three approaches in which we can be proactive in addressing the psychosocial implications of delays in care for head and neck oncology patients:

1. Clear communication from providers
2. Psychosocial assessment
3. Connection to further resources and support groups

## 3. Communication and Shared Decision Making

Clear and transparent communication with patients regarding potential delays in care delivery during the pandemic is paramount. Patients may experience feelings of abandonment or fear due to delays caused by triage decisions and resource constraints. A recent survey by Amaniera et al. assessed psychosocial effects of the pandemic on cancer patients, survivors, and caregivers. The majority of patients included in this study reported social isolation and an impact on mental health due to the pandemic [12]. Patients expressed added stress due to potential lack of access to cancer treatments, and anxiety regarding how resource constraints will impact optimal oncologic care. This is particularly challenging for the head and neck population, given its disproportionate incidence of isolation and limited social support [13,14]. The effects of head and neck cancer and its treatment can impact an individual’s ability to communicate and eat, two important functions for social interaction. Further, surgical treatment for head and neck cancer can lead to facial disfigurement or changes in appearance, which can perpetuate feelings of isolation [13]. Delays in care may further exacerbate these sentiments. Clarity in communication from providers may help alleviate some of these anxieties and help shape how patients perceive their care management. 

While decisions may be governed by guidelines and triage systems that supersede the individual physician, the physician–patient relationship remains the central tenet of patient care. Talking to patients about how their care may be delayed and explaining triage level decisions beyond the control of the individual physician requires a customized approach for each patient. Physicians should convey information to their patients effectively and empathetically. Providers must clearly understand the rationale of their institution’s triage system (which is likely dynamic), so that patients receive a clear and consistent message from all those involved in their care.

Shared decision making between patients and providers has been vital during the pandemic as we navigate the challenges of delaying cases and prioritizing patients equitably [5,15,16,17]. The pandemic has required alteration of treatment paradigms such that patients may no longer be offered a choice of interventions with equivalent outcomes for certain cancers. For example, individuals with surgically resectable head and neck cancer may be offered radiation due to the non-invasive nature of this treatment modality. Literature suggests that shared decision making may encourage patients to address end-of-life decisions and prompt goals of care discussions which may otherwise go unaddressed [18,19]. While these discussions can be overwhelming and challenging for many patients, we must be able to identify patients in need of more structured support. Patients with aggressive diseases may experience a rapid progression of symptoms; therefore, it is crucial to address care plans in light of potential delays in treatment.

## 4. Psychosocial Assessment: Implications of Delays in Care

The psychosocial effects of a head and neck cancer diagnosis are broad. Patients may experience a range of conflicting emotions, including fear, anxiety, depression, anger, and confusion [20,21]. Patients may struggle with coping with the initial diagnosis, engaging in shared decision making, and managing the physical effects of treatment. Information processing and complex decision making regarding treatment can be overwhelming for patients. Patients who have completed oncologic treatment are often still burdened by anxiety and fear of cancer recurrence, functional changes and disfigurement, and chronic pain [22,23,24]. The Diathesis Stress model is a psychological theory that mental health disorders develop from a biologic predisposition in combination with stressful conditions or environment [25]. Head and neck cancer patients already suffer from the stress of their disease. The additive stress of the COVID-19 pandemic further exacerbates their vulnerability for developing psychological distress and mental health disorders.

From a biologic standpoint, the mechanism of action of COVID-19 infection may provide a link between stress, cancer, and COVID-19 [26]. The causative agent for COVID-19, SARS-CoV2, enters the body through interaction with the angiotensin-converting enzyme 2 (ACE2) receptor. This receptor is involved in the Renin Angiotensin System (RAs) which is known to influence both cancer and stress. A recent review by Iftikhar et al. suggests that COVID-19, stress, and cancer may interact mechanistically through RAS to facilitate progression of disease and stress [26].

Studies have illustrated the psychological detriment of COVID-19 for the general population, with an increase in prevalence of depressive symptoms amongst U.S. adults during the pandemic [27]. Even prior to the COVID-19 pandemic, head and neck cancer patients faced significant psychosocial distress due to their diagnosis, treatment, and prognosis. Levels of anxiety and depression have been shown to be higher in head and neck cancer patients than other oncological populations at baseline [23]. The additional stressor of a delay in care during the pandemic may exacerbate emotions of fear, anxiety, and distress [23,28,29]. During the pandemic, psychological distress can impact a patient’s decision regarding their care and treatment. The most common identified causes for psychological distress in cancer patients have been fear of contracting COVID-19, fear of disease progressions, interruption of oncology care, and immunocompromised status [29]. Some research has even suggested that anxiety related to COVID-19 may be so crippling for cancer patients that they may refuse treatment due to fear of infection [29]. Patients’ decision-making processes during the pandemic can be affected by these added psychological stressors in addition to the baseline distress that accompanies a cancer diagnosis [29]. Proactive psychosocial assessments should be integrated into the care of all head and neck oncology patients, especially those whose treatment is affected by triage-level decisions. Such assessments can be conducted remotely through virtual care visits and telemedicine. 

The first step in addressing the psychosocial burden placed on head and neck cancer patients during this time involves respect and acknowledgment of each patient’s unique situation and validation of each patient’s experience. Delayed recognition of psychological distress in cancer patients is associated with poorer quality of life and survival outcomes [30]. The National Comprehensive Cancer Network (NCCN) clinical practice guidelines emphasizes ongoing screening for distress as a key component of patient-centered oncologic care [31]. The NCCN’s distress thermometer and problem checklist should be employed to evaluate all patients undergoing oncologic care and may be especially useful during the pandemic. Other screening tools, such as the Edmonton Symptom Assessment System (ESAS) and the Canadian Problem Checklist, have been shown as effective measures in screening for distress in cancer patients [32,33]. Frequent utilization of these validated tools can help identify patients with elevated psychosocial concerns who may warrant additional support. NCCN recommends that distress screening should be initiated at pivotal medical visits, described as encounters during which patients may be more vulnerable to experiencing distress [23,29]. One strategy may be to assess patients’ responses during notification of treatment delays and any subsequent waiting period prior to initiation of treatment. While head and neck cancer providers cannot bear the responsibility of remedying the psychosocial issues of their patients, they can assist in discerning which patients warrant further resources and support.

## 5. Patient Support: Connection to Further Resources

The importance of psychosocial care for cancer patients has been well-established due to the vulnerability of this population [20,34,35]. With the social isolation and constant uncertainty of the pandemic, many patients may experience exacerbation of psychosocial distress. It is our duty as a medical community to provide psychosocial support for our patients who experience delays in care during the pandemic. In a recent survey of patients and survivors of head and neck cancer designed to elucidate needs and concerns regarding their cancer during the pandemic, the authors found that the most common concern was contraction of COVID-19 [36]. Most respondents reported that they sought emotional support outside of their friends and family, including cancer support groups and online communities [36]. This finding highlights the need for structured and accessible support mechanisms for patients and survivors during this time.

Underserved populations already face significant barriers in access to oncologic care and the COVID-19 pandemic may further exacerbate these inequities [37]. Limitations on in-person interactions especially impact those with low health literacy and limited English proficiency [38]. The advent of telemedicine and virtual healthcare has allowed the opportunity of virtual support groups and virtual counseling. This is one avenue which should be further explored for head and neck oncology patients. While telemedicine has broadened the possibilities for patient contact during the pandemic, we recognize that many patients may not have technological means for video-based community. Others may find telemedicine impersonal, lacking the human connection often gained from in-person communication. Research has shown that older populations, particularly Black and Hispanic patients, are less likely to utilize virtual healthcare [39]. 

In head and neck oncology, many patients may be limited in verbal communication due to the consequences of their cancer and its treatment, which must also be considered when designing effective psychosocial interventions for this patient population. We must prevent further exacerbation of existing disparities by implementing care strategies through an equity lens and improving equitable access to care.

We should develop and hone systems to support patients when delays in care are unavoidable. Creation of facilitator-led support groups may ensure patients have an outlet to express and share their concerns surrounding their care. Having an informed healthcare provider at such meetings would be essential to help dispel untruths and provide reassurance. Integrative models that address biological, psychological, and social factors that impact a patient’s well-being are critical in head and neck oncology. Engaging family members in support groups and developing peer support groups specific to those experiencing care delays may prove beneficial for patients and their families. 

While the pandemic has had devastating effects on our patients, we must also recognize the effects on oncology providers and our healthcare workforce. It is essential to address burnout in healthcare providers in order to optimize care for our patients [40]. Elements of burnout, such as emotional exhaustions, cynicism, and depersonalization are already prevalent amongst oncology providers. The intensified occupational stress during the pandemic further exacerbates the elements of burnout and creates psychological distress for providers [40]. A supportive climate is necessary to ensure that healthcare providers can effectively continue caring for oncology patients.

## 6. Emergence of a New Era

As the pandemic continues to evolve, we must ensure that we are proactively addressing the psychosocial needs of head and neck oncology patients by clearly communicating triage-level decisions, identifying those in need of further psychosocial support, and connecting patients with appropriate resources and programs. Provider awareness is a necessary prelude to identification of patients in need of psychosocial support. Further research is needed to elucidate the impact of triage on the wellness of both providers and patients. The impact of how we address the psychosocial needs of head and neck cancer patients will outlast the pandemic, and thus we must be conscientious in our efforts to provide the highest level of comprehensive care for our patients.
